# Representation of Surgery in Registered Clinical Trials for Brain Metastases: An Analysis of the ClinicalTrials.gov Database

**DOI:** 10.3390/cancers18213507

**Published:** 2026-10-31

**Authors:** Sandra Leskinen, Nathan A. Shlobin, Roland A. Scott, Hannah Haile, Mommina Ashaque, Brian J. A. Gill

**Affiliations:** 1 State University of New York Downstate College of Medicine, 445 Lenox Rd, Brooklyn, NY 11203, USA; sandra.leskinen@downstate.edu (S.L.); ma4117@columbia.edu (M.A.); 2 Department of Neurosurgery, The Neurological Institute of New York, Columbia University Irving Medical Center, 710 W 168th St, New York, NY 10032, USA; nas2227@cumc.columbia.edu (N.A.S.); ras2353@cumc.columbia.edu (R.A.S.); hsh2135@cumc.columbia.edu (H.H.)

**Keywords:** clinical trials, neurosurgery, neuro-oncology, radiotherapy, brain tumor, brain metastases, ClinicalTrials.gov

## Abstract

**Background/Objectives:** Brain metastases (BMs) represent a significant clinical challenge affecting 10–26% of patients who die from cancer. While surgery plays an established role in the management of selected patients, its representation within the registered clinical trial landscape has not been systematically characterized. **Methods:** In this cross-sectional registry analysis, ClinicalTrials.gov was searched on 20 December 2025, using condition terms related to BMs combined with the Boolean operator OR. Interventional and observational studies were included without restrictions on phase, status, sex, or age; trials not updated since 1 January 2015 were excluded. Included studies were classified as surgery-focused, surgery-enabled, or non-surgical. Data were extracted on study characteristics, interventions, and outcomes. **Results:** A total of 493 trials were included. Of these, 57 (11.6%) involved surgery, including 9 (1.8%) surgery-focused and 48 (9.7%) surgery-enabled trials, whereas 436 (88.4%) were non-surgical. Radiotherapy was the dominant treatment platform, present in 262 trials (53.1%), with stereotactic radiosurgery reported in 179 (36.3%). Among the 57 surgical trials, the most common primary endpoint categories were periprocedural/safety and local control outcomes (each 26/57, 45.6%) and survival (20/57, 35.1%). Only 4 surgical trials (7.0%), all surgery-focused, had a primary endpoint that directly measured an operative outcome. **Conclusions:** Surgery accounted for a minority of registered brain metastasis trials and more commonly served as context for evaluating adjuvant, radiation-based, and multimodality approaches than as the primary intervention under study. Surgical trials were weighted toward safety, local control, and survival endpoints, with less frequent emphasis on surgery-specific and functional outcomes.

## Simple Summary

Brain metastases are tumors that have spread to the brain from other parts of the body and represent one of the most challenging problems in cancer care. While surgery plays an important role in the treatment of selected patients, it is not well characterized how often surgery is the focus of dedicated research studies. This study examined all clinical trials for brain metastases registered in ClinicalTrials.gov to understand how surgery is represented in this research landscape. We found that surgery was involved in fewer than one in eight trials, and in most of those cases it served as a platform for testing other treatments, such as radiation or targeted therapy, rather than as the primary focus of investigation. These findings suggest that future trials should place greater emphasis on measuring outcomes that are most relevant to surgical patients, including neurologic recovery and functional independence.

## 1. Introduction

Brain metastases (BMs) represent a significant clinical challenge, developing in 10–26% of patients who die from cancer [1]. Their management is often complex and requires a multidisciplinary approach that addresses both local control of intracranial disease and systemic progression of the underlying malignancy [2]. Advances in systemic therapy have improved survival for many patients with cancer and, in doing so, have increased the clinical significance of BMs by allowing more patients to live long enough to develop intracranial metastatic disease, particularly those with lung cancer, breast cancer, and melanoma [3,4,5].

Current treatment strategies for BMs include surgical resection, stereotactic radiosurgery (SRS), whole-brain radiation therapy (WBRT), and various systemic therapies, including chemotherapy and immunotherapies [2]. Surgery remains an important component of BM care, particularly for patients with limited lesions, large symptomatic tumors, mass effect, or the need for histologic diagnosis [6]. Candidacy for resection also depends on the status of systemic disease, with surgery generally favored when extracranial disease is controlled or has effective treatment options and the patient has an adequate performance status [6,7]. Surgical resection can provide immediate relief of mass effect and neurologic symptoms, and randomized data support improved survival in selected patients with a single BM treated with resection followed by radiotherapy [7,8,9]. Despite its established role in patient care, surgery may be less visible as a primary investigational focus in the trial literature than other treatment modalities because it is often incorporated into multimodality protocols that primarily emphasize radiotherapy, systemic therapy, or translational endpoints rather than the operation itself [10].

The purpose of this study was to characterize the representation of surgery within the registered clinical trial landscape for patients with BMs. Specifically, we sought to determine how often surgery appeared in contemporary BM trials, characterize whether surgery functioned as the primary intervention or as an enabling component of another investigational strategy, and evaluate the characteristics of trials involving surgery against those of non-surgical trials. By situating surgery within the broader BM trial landscape, this study aimed to describe current research patterns and clarify how surgery is represented within evolving multidisciplinary BM care.

## 2. Materials and Methods

### 2.1. Study Collection

**Figure 1:**
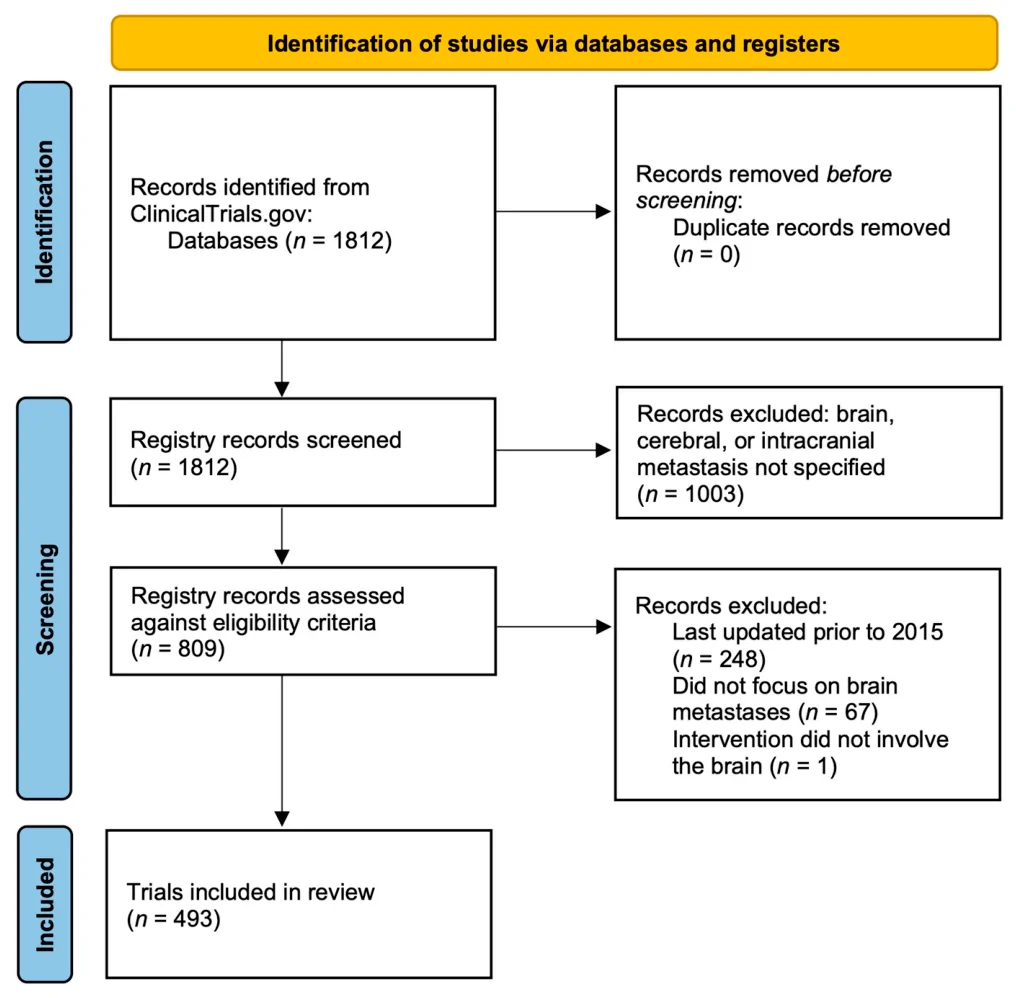
Flow diagram of trial identification, screening, and classification. Records were identified from ClinicalTrials.gov on 20 December 2025 using brain metastasis condition terms combined with OR. Included trials were classified by the role of surgery as surgery-focused, surgery-enabled, or non-surgical – 9 were surgery-focused, 48 were surgery-enabled, and 436 were non-surgical.

ClinicalTrials.gov (National Library of Medicine) was queried on 20 December 2025. The search was filtered by condition/disease terms related to BMs including the following: “brain metastasis,” “brain metastases,” “cerebral metastasis,” “cerebral metastases,” “intracranial metastasis,” and “intracranial metastases”. These terms were entered in the Condition/Disease field and combined with the Boolean operator OR (“brain metastasis OR brain metastases OR cerebral metastasis OR cerebral metastases OR intracranial metastasis OR intracranial metastases”); no additional search filters were applied, and all retrieved records were exported for screening. The NCT identifiers of all included trials are listed in Table S1. The search was designed to capture the full clinical trial landscape of BMs, including studies in which surgery was the primary intervention, an enabling procedural component, or not part of the investigational design. Both interventional and observational studies were included without restrictions on study phase, study start date, recruitment status, sex, or age. The study selection process is summarized in Figure 1. As this is a cross-sectional analysis of a trial registry rather than a systematic review of published evidence, risk-of-bias assessment and quantitative evidence synthesis were not performed. This analysis was not prospectively registered.

### 2.2. Study Selection

Trials were screened by title, conditions, and brief summary. Studies were excluded if they:

1. Included mixed populations of BMs and primary brain tumors or other non-metastatic disease states;
2. Used nonspecific terminology such as “brain tumor,” “intracranial tumor,” “CNS neoplasm,” or “brain neoplasm” without explicit reference to metastatic disease;
3. Did not specifically address the treatment, biology, or outcomes of metastatic brain disease;
4. Had not been updated since 1 January 2015.

The last-updated criterion was chosen as the most inclusive way to capture records maintained in the registry during the contemporary treatment era, including older trials whose status, results, or publications were updated after 2015; it was not intended to define when a trial was conducted. As this criterion does not correspond to trial initiation or completion, the included cohort was additionally stratified by registered study start date, and a sensitivity analysis was restricted to trials first posted on or after 1 January 2015 (Section 3.8).

### 2.3. Classification of Surgical Involvement

Trials were classified according to whether surgery was the primary intervention under study, an enabling procedural component of the investigational treatment strategy, or unrelated to the trial’s central purpose. Classification was based on the registry title, brief summary, conditions, intervention fields, and primary outcome measures. Trials were classified jointly by three authors (SL, HH, BJAG) through discussion until consensus was reached. As classifications were not made independently, formal inter-rater agreement statistics were not calculated.

Surgery-focused trials were those in which the neurosurgical procedure itself constituted the investigational intervention. These included studies evaluating operative technique, extent of resection, surgical adjuncts such as intraoperative mapping or fluorescence guidance, innovations in operative workflow, or changes to perioperative surgical care tested in patients undergoing resection. In these trials, the primary research question centered on surgical performance, efficacy, safety, or feasibility. For example, NCT04116801 entitled “Evaluation of Resection Quality of Cerebral Metastases Using Fluorescence Guided Surgery: a Prospective Randomised [*sic*] Study” evaluated intraoperative fluorescence-guided resection, which would fall into the surgery-focused category.

Surgery-enabled trials were those in which surgical resection was required to enable delivery or definition of the investigational therapy, although the operation itself was not the intervention under study. In these trials, surgery served as the platform that defined the therapeutic context, target anatomy, or treatment strategy. This category included both intraoperative surgery-dependent therapies, such as brachytherapy implantation, intraoperative radiotherapy, device implantation, or catheter placement for locoregional drug delivery, and postoperative therapies defined by prior resection, such as postoperative SRS, postoperative intensity-modulated radiation therapy (IMRT), cavity-directed radiation, or other adjuvant strategies contingent on surgical removal of disease. For example, NCT04114981 entitled “Single Fraction Stereotactic Radiosurgery Compared with Fractionated Stereotactic Radiosurgery in Treating Patients with Resected Metastatic Brain Disease” evaluated postoperative SRS to the resection cavity, which would fall into the surgery-enabled category.

Non-surgical trials were those in which surgery neither constituted the intervention under study nor was required to enable delivery of the investigational therapy. This included studies in which surgery was permitted as background standard care, optional for symptom control, or unrelated to the investigational mechanism, such as systemic therapy trials, radiation-only trials not contingent on prior resection, and diagnostic or imaging studies. For example, NCT02886585, entitled “Pembrolizumab in Central Nervous System Metastases” evaluated pembrolizumab in patients with BMs; surgery was permitted as background standard of care but was not an investigational component, so the trial would be considered non-surgical.

Decision rules were applied in sequence: (1) if a surgical or ablative procedure (resection, laser interstitial thermal therapy [LITT], or a surgical adjunct) or a change to perioperative surgical care was the investigational intervention or a comparator arm, the trial was classified as surgery-focused; (2) otherwise, if enrollment required planned or completed resection, or the investigational therapy required resected tissue, a resection cavity, or a surgically implanted delivery device, the trial was classified as surgery-enabled; (3) all remaining trials, including those in which surgery was permitted but not required, were classified as non-surgical. Perioperative surgical care referred to management that forms part of the operation itself rather than anticancer treatment. The classification, role of surgery, and classification rationale for each of the 57 surgical trials are provided in Table S4.

### 2.4. Data Extraction

The following data were extracted from ClinicalTrials.gov: National Clinical Trial (NCT) number, study title, acronym, study status, brief summary, study results, conditions, interventions, primary outcomes, secondary outcomes, other outcomes, sponsor, collaborators, sex, age, phase, enrollment, funder type, study type, study design, start date, primary completion date, completion date, first posted date, results first posted date, last update posted date, locations, study documents, and study URL. Registered enrollment (actual for completed trials and anticipated for ongoing trials) and the number of listed study locations were used to describe trial size and setting; trials listing one location were considered single-center. Endpoints were categorized from the primary outcome measures registered on ClinicalTrials.gov rather than from full-text publications; for trials with a linked publication, the full-text article was reviewed only to determine whether it reported trial results or a study protocol. Eligibility criteria were not systematically extracted.

Interventions were categorized as procedure, radiation, drug, biologic, device, diagnostic test, or other. In addition, the study team assigned each trial one or more derived treatment classes (e.g., resection, LITT, SRS, WBRT, chemotherapy, biologic therapy, or imaging) from the full registry record to summarize treatment platforms. These derived classes were descriptive and were not used to classify surgical involvement. Study design variables included allocation, interventional model, masking, and primary purpose. Allocation was classified as randomized, non-randomized, not applicable, or not reported. The interventional model was categorized as parallel, sequential, single-group, factorial, or not reported. Masking was classified as none, single, double, triple, or not reported; when masking was used, the masked party or parties (participant, investigator, outcomes assessor, or care provider) were also recorded. The primary purpose was classified as diagnostic, treatment, prevention, screening, health services research, basic science, other, or not reported. Early phase I and phase I studies were grouped as phase I; phase I/II and phase II studies as phase II; phase II/III and phase III studies as phase III; and phase IV studies as phase IV [11].

### 2.5. Study Type Categorization

All included studies were further classified a priori by the study team, in consultation with the senior author, into the following categories to capture recurring clinical and methodological patterns across the included studies and enable structured comparison of surgical trial types within the broader BM trial landscape:

1. Window-of-opportunity trials, which evaluated pre-treatment interventions before standard therapy to assess tumor response, pharmacokinetics, or other biologic effects [12];
2. Drug-delivery trials, which focused on methods to improve delivery of therapy to BMs, including approaches such as intra-arterial or convection-enhanced delivery [13];
3. Outcomes trials, which evaluated clinical endpoints such as survival, quality of life, or cognitive function, often through comparison of treatment strategies;
4. Adjuvant trials, which investigated therapies administered after surgery to reduce recurrence or improve survival, most commonly postoperative radiation or systemic therapy;
5. Adjunct trials, which evaluated therapies administered alongside surgery or intraoperatively to enhance local treatment, such as intraoperative radiotherapy or brachytherapy;
6. Technique studies, which focused on surgical or radiotherapy methods or tools intended to improve resection accuracy or functional preservation;
7. Diagnostic studies, which evaluated imaging or molecular profiling approaches to improve tumor detection, classification, or response assessment;
8. Radiotherapy-planning studies, which evaluated imaging or adaptive methods for radiotherapy target delineation or treatment planning rather than a therapeutic or diagnostic intervention;
9. Other observational studies, which comprised an observational cohort study of tumor displacement during MRI-guided adaptive radiotherapy.

Each trial was assigned to a single study type, so the categories were mutually exclusive and collectively exhaustive. Linked publications were identified by cross-referencing each trial record with the linked publication in ClinicalTrials.gov and confirming PubMed indexing, and were categorized as reports of trial results or study protocols. Publications not linked to the registry record were not sought; therefore, these counts represent registry-linked publications rather than publication rates, and the findings of published trials were not graded as positive or negative.

### 2.6. Endpoint Categorization

Primary endpoints were categorized only for surgery-focused and surgery-enabled trials, as these were the studies in which surgery was either the intervention under study or a required component of therapeutic delivery. Endpoint categorization was based on information reported in the ClinicalTrials.gov registry, which may not reflect the endpoints as ultimately measured or reported in publications. Registry-reported endpoints may be incomplete, inconsistently worded, or not updated following protocol amendments. Endpoint categories were not mutually exclusive, meaning one trial could contribute to more than one category. Primary endpoints were grouped as follows:

1. Survival, including overall survival, progression-free survival, and disease-free survival;
2. Local control, including local control and local progression;
3. Cognitive/patient-reported, including cognitive function, memory, and quality of life assessments;
4. Radiologic, including magnetic resonance imaging (MRI)-based tumor response, positron emission tomography (PET) imaging outcomes, and lesion changes on imaging;
5. Periprocedural/safety, including adverse events, periprocedural complications, and treatment-related toxicities;
6. Surgical, encompassing the efficacy, safety, and impact of surgical interventions or surgically defined treatment strategies, including tumor resection extent, complications, functional recovery, and recurrence rates [14];
7. Drug delivery/pharmacokinetics, including feasibility of drug delivery methods, pharmacokinetic studies, and biomarker levels related to treatment response.

Each registered primary outcome measure was assigned to every category it addressed, so endpoints such as treatment-related toxicity could be coded as both periprocedural/safety and surgical. To distinguish endpoints that directly measure the operation from outcomes attributed to a surgical strategy, endpoints coded as surgical were further classified as operative-specific when they measured extent of resection, ablation completeness, or postoperative neurologic or functional status. Endpoints were reported separately for surgery-focused and surgery-enabled trials.

## 3. Results

### 3.1. Search Results

A total of 1812 trials were identified in the initial search. Trials that did not specify brain, cerebral, or intracranial metastasis in the title, brief summary, or condition fields were excluded (*n* = 1003). Studies that were last updated prior to the year 2015 were similarly removed from analysis (*n* = 248). An additional 67 studies were excluded because they did not focus on BMs. One study was excluded because the intervention involved breast surgery rather than brain surgery. In total, 493 studies were included for analysis (Figure 1; Table 1).

### 3.2. Trial Characteristics

Of the 493 included trials, 443 (89.9%) were listed as interventional, while 50 (10.1%) were observational. With respect to study status, the majority were either recruiting (126/493, 25.6%) or had been completed (126/493, 25.6%), while a smaller proportion were listed as active but not recruiting, terminated, withdrawn, or with unknown status. Most studies enrolled both male and female participants (458/493, 92.9%), with a small subset restricted to female participants only (35/493, 7.1%).

In terms of trial phase, 53 studies were categorized as Phase I, 228 as Phase II, 51 as Phase III, and 5 as Phase IV. A subset of 156 trials did not report a specific phase designation (Table S1).

The most commonly represented primary tumor types were non-small cell lung cancer (103/493, 20.9%), breast cancer (87/493, 17.6%), and melanoma (40/493, 8.1%). Twenty-five studies (5.1%) focused exclusively on recurrent BMs, 29 (5.9%) on newly diagnosed disease, and 15 (3.0%) on large BMs.

Excluding 23 withdrawn trials with zero enrollment, the median registered enrollment was 46 participants. Overall, 300 trials (60.9%) listed a single study location, 151 (30.6%) listed two or more locations, and 42 (8.5%) did not report locations.

### 3.3. Study Design Features

Among the 443 interventional trials, 207 explicitly reported allocation as randomized or non-randomized: 74.9% (155/207) were randomized, while 25.1% (52/207) were non-randomized. The most common interventional models were the single-group model and parallel model, utilized in 58.0% (257/443) and 39.3% (174/443) of studies, respectively. Other models included sequential (10/443; 2.3%) and factorial (1/443; 0.2%) designs; the interventional model was not reported for 1 trial. Masking was not implemented in 92.1% of interventional trials. In those that did employ masking (7.9%), single, double, or triple masking approaches were used.

The primary purpose of most interventional trials was treatment (378/443, 85.3%), followed by diagnostic (33/443, 7.4%) and supportive care (12/443, 2.7%) aims. A smaller number of studies pursued objectives related to prevention (6/443, 1.4%), device feasibility (3/443, 0.7%), screening (2/443, 0.5%), or health services research (1/443, 0.2%), or were otherwise unspecified (8/443, 1.8%) (Table S1).

### 3.4. Interventions Across Trial Landscape

Radiotherapy (RT) alone was the most common intervention category, accounting for 110 trials (22.3%), followed by imaging (48, 9.7%), RT plus biologic therapy (47, 9.5%), biologic monotherapy (44, 8.9%), and combination biologic therapy (40, 8.1%). Resection plus RT accounted for 27 trials (5.5%), followed by chemotherapy plus biologic therapy (22, 4.5%), RT plus small molecule therapy (14, 2.8%), and RT plus chemotherapy (13, 2.6%). When interventions were decomposed into component treatment classes, RT was the dominant therapeutic platform, present in 262 of 493 trials (53.1%). Biologic therapies appeared in 191 trials (38.7%), followed by chemotherapy in 70 (14.2%), imaging-based approaches in 54 (11.0%), and resection or LITT in 37 (7.5%). The operative treatment class and the registered intervention fields reflect different definitions. Treatment classes were assigned by the study team from the registry record, whereas the ClinicalTrials.gov intervention fields only contain the interventions entered by the sponsor. Notably, the 37 trials with resection of LITT as a derived treatment class are distinct from the 57 trials classified as surgical. This discrepancy reflects the fact that many surgery-enabled trials incorporated surgery as context for another investigational therapy rather than listing it as a formal intervention, and thus surgery did not always appear in structured intervention fields even when it was a required component of the study design. At the trial level, only 14 of the 57 surgical trials (24.6%) listed a surgical or ablative procedure in the registered intervention fields. In the remaining 43, the role of surgery was identified from the title, brief summary, or outcomes descriptions (Table S4).

**Figure 2:**
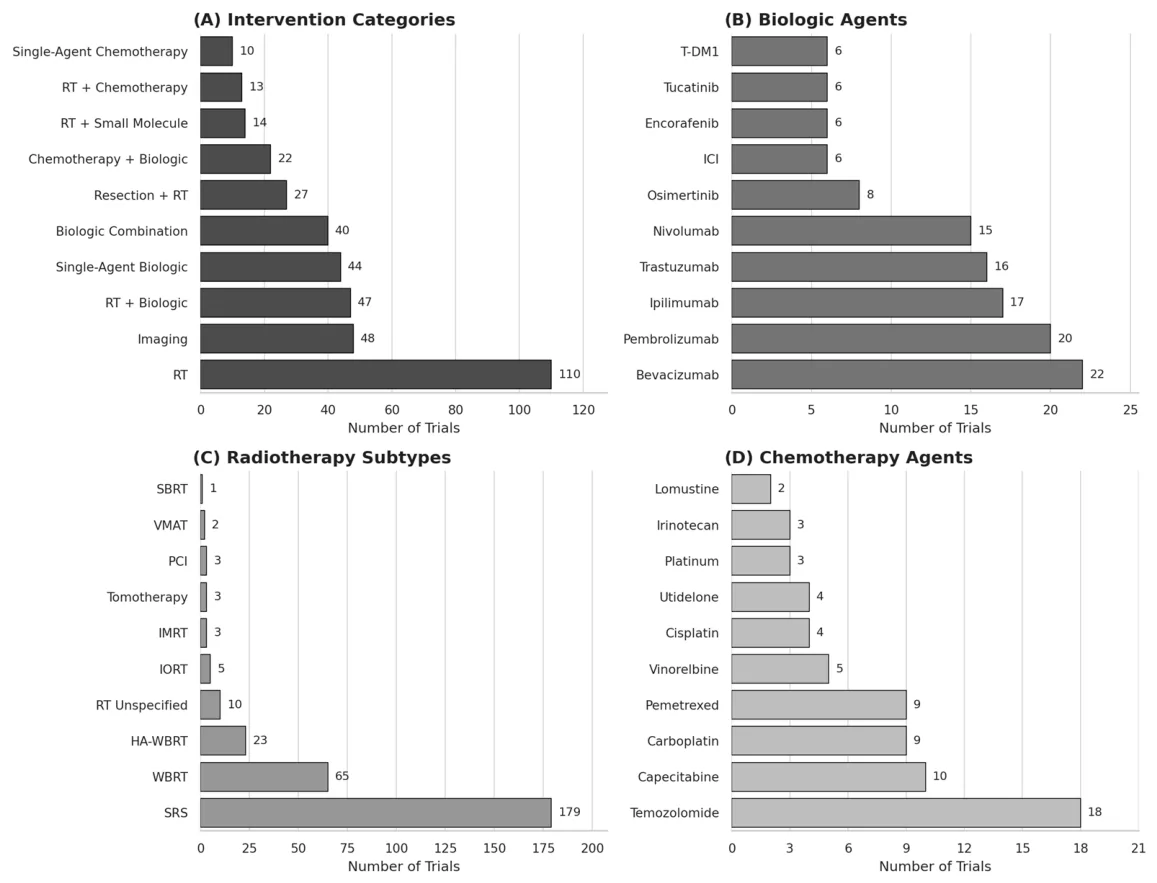
Distribution of intervention categories and commonly studied treatment subtypes across included clinical trials. (**A**) Most frequent intervention categories. (**B**) Most common biologic agents among biologic-containing trials. (**C**) Most common radiotherapy subtypes among radiotherapy-containing trials. (**D**) Most common chemotherapy agents among chemotherapy-containing trials. Abbreviations: RT—Radiotherapy; ICI—Immune Checkpoint Inhibitors; SRS—Stereotactic Radiosurgery; WBRT—Whole-Brain Radiotherapy; HA-WBRT—Hippocampal-Avoidance WBRT; IORT—Intraoperative Radiotherapy; IMRT—Intensity-Modulated Radiotherapy; PCI—Prophylactic Cranial Irradiation; VMAT—Volumetric Modulated Arc Therapy; SBRT—Stereotactic Body Radiotherapy.

Among RT-containing studies, SRS was by far the most common modality, appearing in 179 trials, corresponding to 36.3% of all trials and 68.3% of RT-containing trials. WBRT appeared in 65 trials (13.2% of all trials; 24.8% of RT-containing trials), and hippocampal-avoidance WBRT in 23 (4.7% of all trials; 8.8% of RT-containing trials). Overall, the trial landscape was dominated by RT-centered designs, particularly SRS-based approaches, whereas chemotherapy- and biologic-containing studies were more heterogeneous and often incorporated as adjunctive systemic therapies (Figure 2).

### 3.5. Study Type Categorization and Publication Status

By predefined study type, 361 of 493 trials (73.2%) were categorized as outcomes studies, 61 (12.4%) as diagnostic studies, 27 (5.5%) as window-of-opportunity studies, 22 (4.5%) as technique studies, 11 (2.2%) as adjuvant studies, 6 (1.2%) as adjunct studies, 2 (0.4%) as drug-delivery studies, and 3 (0.6%) as radiotherapy-planning (*n* = 2) or other observational (*n* = 1) studies (Table S2). Overall, 52 of 493 trials (10.5%) had a ClinicalTrials.gov-linked, PubMed-indexed publication. Of these, 42 reported trial results and 10 (19.2%) were study protocols. Since this analysis was limited to publications indexed in PubMed and linked to ClinicalTrials.gov records, this proportion reflects registry linkage rather than a true publication rate.

### 3.6. Surgical Trials

**Figure 3:**
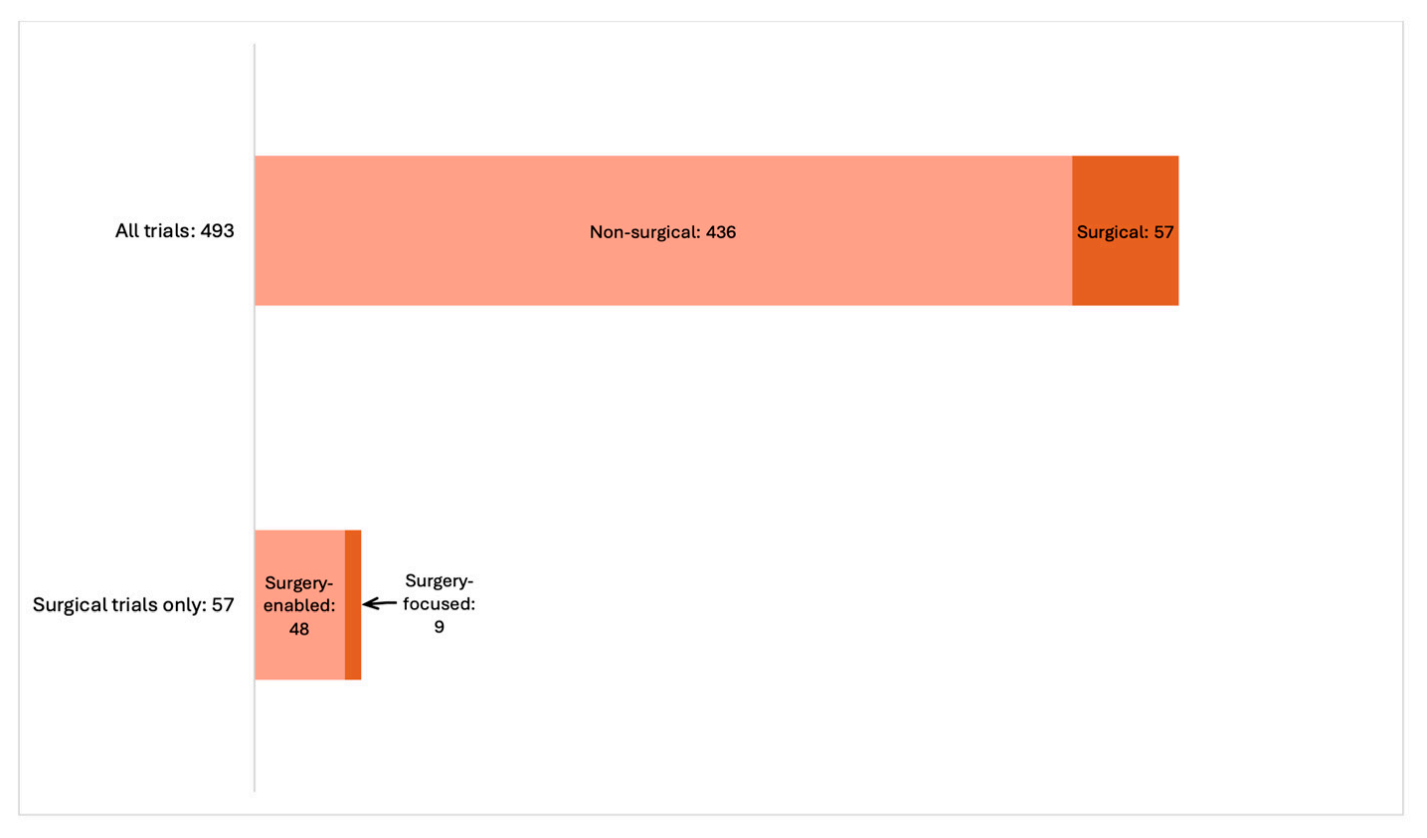
Representation of surgery within the overall clinical trial landscape for brain metastases. Of 493 included trials, 57 involved surgery. Within this subset, surgery was more commonly incorporated as an enabling component of multimodality treatment than studied as the primary investigational intervention.

Of the 493 included trials, 57 (11.6%) involved surgery, including 9 surgery-focused and 48 surgery-enabled trials; the remaining 436 (88.4%) were non-surgical (Figure 3). Of the 9 surgery-focused trials, 8 were interventional and 1 was observational. Four were categorized as technique studies and 5 as outcomes studies. None had a ClinicalTrials.gov-linked, PubMed-indexed publication at the time of data extraction. Among the 8 interventional surgery-focused trials, allocation was randomized in 4, non-randomized in 1, and not reported in 3. Five used a parallel and 3 used a single-group interventional model. Their interventions included resection, laser interstitial thermal therapy (LITT), intraoperative MRI-guided resection, intraoperative sodium fluorescein, perioperative dexamethasone, and resection- or LITT-based approaches evaluated with adjunctive SRS, hypofractionated stereotactic radiotherapy, or pembrolizumab.

Among the 48 surgery-enabled trials, 47 were interventional and 1 was observational. These were categorized as window-of-opportunity studies (24), followed by adjuvant (11), adjunct (6), outcomes (6), and technique (1) studies. Ten had a linked PubMed-indexed publication and 38 did not. Among the 47 interventional surgery-enabled trials, allocation was randomized in 24, non-randomized in 5, and not reported in 18; 24 used a parallel, 21 a single-group, and 2 a sequential interventional model. Median registered enrollment was 98 for surgery-focused and 51 for surgery-enabled trials, compared with 44 for non-surgical trials, and 5 of 9 surgery-focused and 28 of 48 surgery-enabled trials listed a single study location. Their interventions most commonly involved perioperative radiation-based approaches, particularly preoperative or postoperative SRS, with additional studies evaluating intraoperative radiotherapy (IORT), hypofractionated IMRT or stereotactic radiotherapy (SRT), Cesium-131 (Cs-131) brachytherapy, GammaTile, Gliadel, perioperative or neoadjuvant systemic therapies, intraventricular cellular therapies, and pharmacokinetic or drug-sensitivity platforms (Table 2).

### 3.7. Primary Endpoints and Publication of Surgical Trials

Across the 57 trials involving surgery, the most common primary endpoint categories were periprocedural/safety (26/57, 45.6%), local control (26/57, 45.6%), and survival (20/57, 35.1%). Surgical outcomes were included in 11 trials (19.3%), radiologic in 7 (12.3%), cognitive/patient-reported in 4 (7.0%), and drug delivery/pharmacokinetic outcomes in 4 (7.0%) (Table S3). Because endpoint domains were not mutually exclusive, totals across categories exceed the number of surgical trials. When analyzed separately, surgery-focused trials most often used surgical endpoints (6/9), whereas surgery-enabled trials most often used local control (23/48) and periprocedural/safety (23/48) endpoints, followed by survival. Of the 11 trials with a primary endpoint coded as surgical, 4 (7.0% of all surgical trials), all surgery-focused, measured an operative outcome directly. The remaining 7 assessed local control, survival, toxicity, or a composite of central nervous system events attributed to a surgical strategy. Four trials had a primary endpoint coded as both surgical and periprocedural/safety. No surgical trial used performance status as a primary endpoint.

Overall, 10 of 57 surgical trials (17.5%) had a ClinicalTrials.gov-linked, PubMed-indexed publication. Among these, 7 reported trial results and 3 were study protocols.

### 3.8. Analysis by Trial Era and Sensitivity Analysis

When trials were grouped by registered start date, surgical trials accounted for 3 of 31 (9.7%) trials starting in 2000–2009, 6 of 79 (7.6%) in 2010–2014, 16 of 137 (11.7%) in 2015–2019, and 32 of 245 (13.1%) in 2020–2026 (Table 3). Seven of the 9 surgery-focused trials started in 2020 or later. Over the same time periods, the proportion of trials incorporating WBRT declined from 29.0% to 6.1% (*p* < 0.001), whereas the proportion incorporating SRS remained similar (32.3% vs. 36.7%, *p* = 0.97). In a sensitivity analysis restricted to the 389 trials first posted on or after 1 January 2015, 49 (12.6%) involved surgery, including 9 (2.3%) surgery-focused and 40 (10.3%) surgery-enabled trials, similar to the primary analysis.

## 4. Discussion

### 4.1. Overview of Study

This registry analysis characterizes the clinical trial landscape for BMs, with particular attention to the representation and role of surgical interventions. While research in this space is increasingly shaped by advances in systemic therapies and radiotherapy, surgery continues to play an important role in patient care, particularly for those with large symptomatic lesions, uncertain diagnoses, or resectable dominant tumors [7]. This is reflected in current clinical practice guidelines from ASCO, SNO, and ASTRO, which recommend surgery for patients with accessible lesions causing mass effect or neurological compromise, particularly when tissue diagnosis is needed or local control is the primary therapeutic goal [6]. Randomized trials have historically demonstrated the benefit of resection plus WBRT compared with WBRT alone in selected patients with solitary metastases [8,9].

Among 493 included trials, 57 were classified as surgical, indicating that surgery accounted for a minority of the registered trial portfolio. This distribution should not be interpreted as evidence that surgery is underrepresented in an absolute sense, as many BM trials appropriately focus on systemic therapy or radiation-based strategies that reflect the primary research questions for the majority of patients with BMs, particularly those who are not surgical candidates or for whom surgery is not the central clinical question. Within this subset, surgery had heterogeneous roles, ranging from studies directly evaluating operative strategies and surgical adjuncts to studies in which surgery provided the procedural context for perioperative radiation, intraoperative treatment, local drug delivery, or tissue-based investigation. Only 9 of the 57 surgical trials were surgery-focused, including studies of ablation, intraoperative imaging guidance, and comparisons between surgical and radiation-based approaches. None of the surgery-focused studies had a linked publication at the time of data extraction, and they were methodologically heterogeneous, largely emphasizing operative workflow, local treatment integration, and selected clinical niches rather than broad resection-based effectiveness questions. As this group was small and its size depends on registry detail and on the classification framework, its relative scarcity should be interpreted descriptively.

The remaining 48 trials incorporated surgery as an essential component of a multidisciplinary treatment strategy rather than as the primary intervention under study. These surgery-enabled studies most commonly used perioperative radiation-based designs, particularly preoperative and postoperative SRS, and were concentrated in window-of-opportunity and adjuvant trial designs. They also included intraoperative and local therapeutic platforms such as IORT, Cs-131 brachytherapy, GammaTile, and Gliadel, as well as perioperative systemic and translational approaches involving immunotherapy, targeted therapy, chimeric antigen receptor (CAR) T-cell delivery, and tissue-based drug-sensitivity or pharmacokinetic studies. This pattern suggests that neurosurgical innovation in brain metastasis trials is often captured through enabling technologies, surgically delivered therapeutics, and translational platforms that depend on surgical access without making the operation the primary investigational variable [15,16].

Of the 10 surgical trials with linked, PubMed-indexed publications, 3 were protocols, leaving 7 reports of trial results. These reports most often addressed local control, feasibility, and toxicity-related outcomes rather than survival: postoperative cavity SRS improved local control compared with observation, salvage SRS was noninferior to WBRT for overall survival after resection in selected patients, and several preoperative SRS studies reported favorable local control with low toxicity [17,18,19,20,21]. This distribution mirrors the broader intervention profile of the dataset, in which radiotherapy was the dominant therapeutic platform overall and SRS was the most common modality. Within that context, it is perhaps unsurprising that surgery-enabled trials were concentrated largely in perioperative radiation-based designs rather than in surgery-centered comparative studies.

Surgical access may also function as a research platform for tissue acquisition, pharmacokinetic assessment, local therapy delivery, and post-treatment biologic analysis, helping explain why surgery was more often represented as an enabling component of multidisciplinary investigation than as the primary intervention under study [15,22]. In this respect, the findings of the present study align with a broader shift in neuro-oncology trial design, in which the neurosurgeon’s role increasingly extends beyond resection alone toward precision delivery, device-based therapies, image-guided intervention, and early-phase translational investigation [16].

Stratification by start date did not suggest that the surgical share of the trial portfolio declined as SRS replaced WBRT and as systemic therapies with intracranial activity emerged. The proportion of surgical trials was similar across periods, whereas WBRT-containing trials declined sharply. Seven of the 9 surgery-focused trials started in 2020 or later, which may reflect renewed interest in operative adjuncts and ablative techniques such as LITT.

### 4.2. Implications and Challenges of Surgical Trial Design

Despite surgery’s established role in the care of selected patients with BMs, only 4 of 57 surgical trials in the present study used a primary endpoint that directly addressed surgery-specific questions such as extent of resection, ablation completeness, or postoperative functional status. Instead, the endpoint structure of surgical trials was weighted toward local control, survival, and periprocedural or safety outcomes, more closely resembling the structure of non-surgical oncology trials than the operative decision-making questions unique to surgery [21,23,24].

Performance status, typically measured with the Karnofsky Performance Status, is central to surgical candidacy and to interpreting postoperative recovery, yet no surgical trial in this cohort used it as a primary endpoint and only 4 listed it among any registered outcome measures. Since performance status thresholds are usually specified in eligibility criteria, which were not systematically extracted, we could not assess how performance status was used to select patients.

This endpoint pattern likely reflects the practical and methodological challenges of studying surgical interventions prospectively. As described more broadly in the surgical literature, operative trials often face difficulty with accrual, patient and surgeon treatment preferences, maintenance of equipoise, limited feasibility of blinding, learning-curve effects, inter-surgeon variability, and challenges in standardizing procedural quality across institutions [25,26,27]. These barriers are especially relevant in BM care, where candidacy for surgery depends on lesion size, number, location, symptom burden, mass effect, extracranial disease status, and the need for tissue diagnosis, and where urgent clinical circumstances may narrow the window for enrollment and randomization.

More broadly, specialty-specific structural factors may also contribute to this pattern. In neuro-oncology, innovation may be driven through collaborative, translational, and technology-enabled studies, while formal prospective trial leadership may also be limited by infrastructure demands, protected research time, and the complexity of organizing surgical trials across institutions [15]. These factors may further contribute to why neurosurgical innovation is often captured through collaborative or platform-based studies rather than through surgery-centered comparative trials alone [15,16].

Taken together, these findings suggest that future work in this area may benefit from greater attention to endpoints that better capture the specific contribution of surgery within contemporary multidisciplinary care, including postoperative neurologic function, resection quality, steroid dependence, patient-reported outcomes, functional independence, and longer-term cognitive outcomes. The present dataset also suggests that surgery is more often positioned as an enabling platform within modern BM trials than as the isolated object of investigation itself, particularly in studies involving perioperative radiation, local therapeutic delivery, and window-of-opportunity tissue-based research [16,22].

### 4.3. Limitations

We acknowledge several limitations to this study. First, only ClinicalTrials.gov was queried, which may have omitted relevant trials registered in other international registries, such as the WHO International Clinical Trials Registry Platform, EU Clinical Trials Register, or other regional registries, and trials registered only under a primary cancer condition without a brain metastasis term may have been missed. The search strategy was also not validated against a reference set of landmark brain metastasis trials. Second, ClinicalTrials.gov registry entries vary considerably in their level of detail and completeness; some brain metastasis trials may include relevant surgical components only in protocol documents, eligibility criteria, or narrative descriptions rather than in structured intervention or outcomes fields, which could lead to underestimation of the surgical trial landscape. Third, publications were identified only through ClinicalTrials.gov links and PubMed indexing. Linked-publication counts therefore likely underestimate true publication, and the findings of published trials were not appraised. Fourth, the analysis relied on registry-reported information, and structured trial fields may not have fully captured the therapeutic role of surgery within multimodality protocols. This was reflected in the discrepancy between trials that explicitly listed surgery as an intervention and those in which surgery was described narratively or functioned primarily as the context for another therapy. Fifth, the framework used to distinguish surgery-focused, surgery-enabled, and non-surgical studies was developed by the study team to characterize the trial landscape, was not externally validated, and remains interpretive. Sixth, the included studies were heterogeneous in design, phase, status, and outcome structure, which limits direct comparison across trials. Seventh, registry records do not consistently report attrition, losses to follow-up, or actual follow-up duration. Registered primary outcome assessment windows ranged from within 48 h of surgery to 15 years, and enrollment for ongoing trials reflects anticipated rather than actual accrual. Eighth, the last-updated criterion may have excluded older completed trials that were not updated after 2015. Finally, no formal assessment of publication bias, trial quality, or risk of bias was performed.

## 5. Conclusions

Surgery represented a minority of registered clinical trials for brain metastases and was more often used as the procedural context for multimodality treatment than studied as the primary intervention itself. Surgical trials most commonly emphasized safety, local control, and survival, with less frequent attention to surgery-specific and functional outcomes. More consistent registry reporting and greater attention to outcomes relevant to operative decision-making and postoperative recovery may help better define the contribution of surgery within contemporary brain metastasis care. Future registry-based and prospective studies should incorporate standardized surgery-specific outcomes, including extent of resection, postoperative neurological status, functional independence, corticosteroid dependence, patient-reported outcomes, and cognitive function.

## Figures and Tables

**Table 1:** Summary of the overall registered clinical trial landscape for brain metastases. Values are presented as counts with percentages where applicable. Radiotherapy was the dominant treatment platform overall, and surgery was represented in a minority of trials.

Characteristic	*N* (%)
Total included trials	493
Interventional	443 (89.9)
Observational	50 (10.1)
Surgical trials	57 (11.6)
Non-surgical trials	436 (88.4)
Linked PubMed-indexed publication	52 (10.5)
Status	
Recruiting	126 (25.6)
Completed	126 (25.6)
Active, not recruiting	45 (9.1)
Other	196 (39.8)
Phase	
I	53 (10.8)
II	228 (46.2)
III	51 (10.3)
IV	5 (1.0)
Not reported	156 (31.6)
Primary cancer	
NSCLC	103 (20.9)
Breast	87 (17.6)
Melanoma	40 (8.1)
Representative treatment classes	
Radiotherapy	262 (53.1)
Biologic therapy	191 (38.7)
Chemotherapy	70 (14.2)
Imaging-based	54 (11.0)
Resection or LITT	37 (7.5)

**Table 2:** Comparison of surgery-focused and surgery-enabled clinical trials for brain metastases. Surgery-focused trials directly evaluated operative interventions or surgical adjuncts, whereas surgery-enabled trials incorporated surgery as a required procedural platform for perioperative, local, or translational therapeutic investigation.

Characteristic	Surgery-Focused Trials	Surgery-Enabled Trials
Total trials	9	48
Interventional	8	47
Observational	1	1
Linked PubMed-indexed publication	0	10
No linked publication	9	38
Most common study types	Outcomes (5), Technique (4)	Window-of-Opportunity (24), Adjuvant (11), Adjunct (6), Outcomes (6)
Common intervention patterns	LITT, iMRI-Guided Resection, Fluorescein-Guided Resection, Perioperative Dexamethasone, Surgery/SRS or Surgery/Immunotherapy Combinations	Timing of SRS, IORT, Brachytherapy, Perioperative Systemic Therapy
Allocation: Randomized	4	24
Allocation: Non-randomized	1	5
Allocation: Not reported	3	18
Dominant endpoint profile	Surgical, Survival, Local Control, Periprocedural/Safety	Local Control, Periprocedural/Safety, Survival

Abbreviations: iMRI—Intraoperative Magnetic Resonance Imaging; IORT—Intraoperative Radiotherapy; LITT—Laser Interstitial Thermal Therapy; SRS—Stereotactic Radiosurgery.

**Table 3:** Registered brain metastasis trials by study start date.

Start Date	Trials, *n*	Surgical, *n* (%)	Surgery-Focused, *n*	Surgery-Enabled, *n*	SRS, *n* (%)	WBRT, *n* (%)
2000–2009	31	3 (9.7)	0	3	10 (32.3)	9 (29.0)
2010–2014	79	6 (7.6)	1	5	28 (35.4)	22 (27.8)
2015–2019	137	16 (11.7)	1	15	50 (36.5)	18 (13.1)
2020–2026	245	32 (13.1)	7	25	90 (36.7)	15 (6.1)
First posted on or after 2015	389	49 (12.6)	9	40	-	-

*p*-values (chi-squared test): surgical, *p* = 0.60; SRS, *p* = 0.97; WBRT, *p* < 0.001. Abbreviations: SRS—stereotactic radiosurgery; WBRT—whole-brain radiation therapy.

## Data Availability

The original data presented in the study are openly available in the ClinicalTrials.gov database at ClinicalTrials.gov.

## Supplementary Materials

The following supporting information can be downloaded at https://www.mdpi.com/article/10.3390/cancers18213507/s1, Table S1: All included studies: data retrieved from the ClinicalTrials.gov database; Table S2: All included trials categorized by study type; Table S3: Characterization of primary endpoints in surgical studies; Table S4: Classification, role of surgery, and rationale for the 57 surgical trials.

## Abbreviations

The following abbreviations are used in this manuscript:

BM	Brain metastasis
SRS	Stereotactic radiosurgery
WBRT	Whole-brain radiation therapy
HA-WBRT	Hippocampal-avoidance WBRT
IMRT	Intensity-modulated radiation therapy
NCT	National Clinical Trial
MRI	Magnetic resonance imaging
iMRI	Intraoperative MRI
PET	Positron emission tomography
LITT	Laser interstitial thermal therapy
RT	Radiotherapy
IORT	Intraoperative radiotherapy
ICI	Immune checkpoint inhibitors
PCI	Prophylactic cranial irradiation
VMAT	Volumetric modulated arc therapy
SRT	Stereotactic radiotherapy
SBRT	Stereotactic body radiotherapy
